# The Jenner Monument

**Published:** 1853-09

**Authors:** 


*The Jenner Monument.*—Collections for the Jenner monument are progressing. The committee acknowledge the following sums received during the past year: United States of America, £339 12*s.* 8*d.*; Russia, £100; Great Britain and Ireland, £153 2*s.* 5*d.* The United States, it is perceived, contribute a handsome proportion.

